# Study of Eddy Current Testing Ability on SLM Aluminium Alloy

**DOI:** 10.3390/ma17143568

**Published:** 2024-07-18

**Authors:** Matúš Geľatko, Michal Hatala, František Botko, Radoslav Vandžura, Jiří Hajnyš

**Affiliations:** 1 Faculty of Manufacturing Technologies, Technical University of Košice with a Seat in Prešov, 080 01 Prešov, Slovakia; matus.gelatko@tuke.sk (M.G.);; 2 Center of 3D Printing Protolab, Department of Machining, Assembly and Engineering Technology, Faculty of Mechanical Engineering, VSB-TU Ostrava, 17. Listopadu 2172/15, 708 00 Ostrava, Czech Republic

**Keywords:** additive manufacturing, selective laser melting, non-destructive testing, eddy current, AlSi10Mg

## Abstract

The detection of defects in aluminium alloys using eddy current testing (ECT) can be restricted by higher electrical conductivity. Considering the occurrence of discontinuities during the selective laser melting (SLM) process, checking the ability of the ECT method for the mentioned purpose could bring simple and fast material identification. The research described here is focused on the application of three ECT probes with different frequency ranges (0.3–100 kHz overall) for the identification of artificial defects in SLM aluminium alloy AlSi10Mg. Standard penetration depth for the mentioned frequency range and identification abilities of used probes expressed through lift-off diagrams precede the main part of the research. Experimental specimens were designed in four groups to check the signal sensitivity to variations in the size and depth of cavities. The signal behavior was evaluated according to notch-type and hole-type artificial defects’ presence on the surface of the material and spherical cavities in subsurface layers, filled and unfilled by unmolten powder. The maximal penetration depth of the identified defect, the smallest detectable notch-type and hole-type artificial defect, the main characteristics of signal curves based on defect properties and circumstances for distinguishing between the application of measurement regime were stated. These conclusions represent baselines for the creation of ECT methodology for the defectoscopy of evaluated material.

## 1. Introduction

The demanding requirements of industrial development include precision and reliability of material inspection within the whole manufacturing process. The necessity of non-destructive testing method application is crucial mainly due to its non-invasive influence on evaluated components. Eddy current testing is a part of surface methods within the identification of electrically conductive materials. Identification itself using this method seems to be simple and fast. However, its incorporation into the quality control of specific material and in specific shapes can be challenging, due to its characteristic parameters, mainly in the form of the lift-off effect, related to the probe-surface variation and indirectly to the shape of the component, edge effect, related to the size of the probe and component, and skin effect, related to the penetration of eddy currents into the material, which all are influenced by excitation frequency, coil characteristics and material properties [1]. Monitoring the influence of the mentioned factors is the actuating impulse for conducting research focused on increasing the ability of ECT application [2]. It includes the design of specific probes [3] and their optimisation for a certain purpose [4], evaluating the possibility of automation, for which the ECT method has predispositions [5], or the application of improved multi-frequency PEC (Pulsed Eddy Current) method [6].

Electrical conductivity, which is the fundamental parameter of the ECT method, can be the critical factor during the identification of high-conductive materials, mainly in the case of discontinuities not reaching the surface of a component, when higher attenuation of the impedance signal occurs. Nevertheless, few experiments have been carried out on aluminium plates using ECT, in terms of coil enhancement, for example, the proposition of “butterfly” double-excitation coils for the intensification of eddy currents during the identification [7] and application of GMR (Giant MagnetoResistance) coil configuration, which showed the possibility of a method for the classification of three crack characteristics (width, depth, orientation) [8], or a customised ECT probe for aluminium parts made using Wire + Arc Additive Manufacturing (WAAM) [9]. Aluminium was also a material of interest for ECT applications as the part of Friction Stir Welding (FSW) butt joints [10] or the honeycomb sandwich structure made by the combination of carbon-fiber-reinforced polymer (CRFP) panels with aluminium [11].

Nowadays, aluminium alloys are widely used within the currently widespread additive manufacturing processes [12]. Eddy current testing was used for the quality assessment of AM materials in a few studies. Sun et al. [13] conducted research focused on porosity measurement in simple SUS 316L stainless steel specimens using an ECT probe, with promising results verified by X-ray tomography for density evaluation. In another study [14], SS 316L stainless steel and titanium alloy Ti-64 were materials of interest during the identification of two types of defects using two different probes and describing the signal-to-defect characteristics and detectability of both probes. The ECT method was also used for the expression of dependency between the relative magnetic permeability variation of maraging 300 steel and cyclic loading, within a complex study of fatigue monitoring investigation [15]. Subsurface defects were identified using ECT in SLM (selective laser melting) Inconel 738LC alloy with a discussion of influencing factors in the form of frequency, the lift-off effect, defect properties, residual stress and roughness [16]. Frequently used AM aluminium alloy AlSi10Mg was investigated using ECT, with the finding of a significant correlation between obtained signal and material conductivity influenced by density variation [17]. Spurek and co-authors [18] used this correlation for a subsequent experiment focused on the in situ identification of density in AlSi10Mg parts layer-by-layer, by mounting the ECT system on an AM machine recoater, with promising results. Valuable results were also obtained during the identification of various densities in AlSi10Mg specimens using an ECT-based HTS-SQUID system for the compensation of sensitivity at low-frequency measurement [19]. A device working on the eddy current principle was used for the measurement of electrical conductivity in heat-treated DMLS (Direct Metal Laser Sintering) AlSi10Mg material, which increased with the application of heat-treatment processes, and its relationship with corrosion resistance was determined [20].

It is generally known that additive manufactured materials do not reach 100% density, which is the parameter that can influence the density of eddy currents. Subsequently, lower density affects the conductivity of the evaluated material, which is the main material parameter influencing the behavior of eddy currents; hence, the sensitivity and standard penetration depth can be different in comparison with conventionally manufactured material. Also, the SLM process causes the formation of different shapes of grains within the microstructure, which is another factor to which eddy currents are susceptible. Complex studies focused on the ECT application within the evaluation of different types of defects in the AM AlSi10Mg aluminium alloy are still missing, which is the main motivation for this research. A described experiment was performed by the reason of checking the ability of the ECT method to identify defects on the surface and in the subsurface layers of the mentioned material, identify maximal possible penetration depth and check the ability of three different probes at the various excitation frequencies. The lift-off effect and skin effect, as important parameters of all probes, were investigated, with subsequent identification of the designed artificial defects, analysis of the obtained ECT device signal characteristics and optimal measurement parameter setting, for the future creation of eddy current testing methodology for the identification of defects in AM materials.

## 2. Materials and Methods

**Figure 1:**
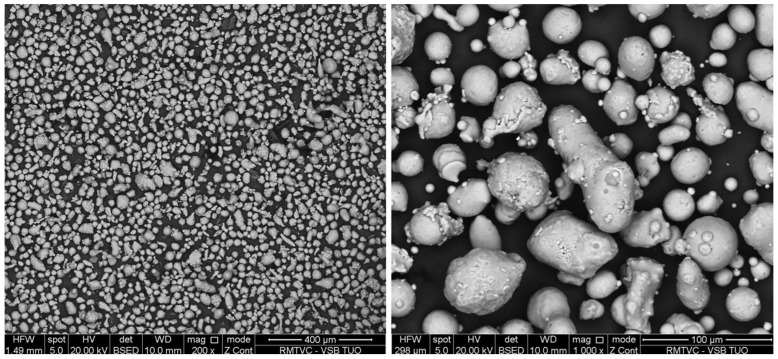
SEM image of aluminium alloy (AlSi10Mg) powder.

The powder material selected for experimental specimens was aluminium alloy AlSi10Mg, with the characteristics of good corrosion resistance, the ratio between strength and mass and heat properties, mainly represented by thermal shock resistance. Its composition is included in Table 1. Particle size distribution of used powder is in the range of 20 to 63 µm (Figure 1).

Additive manufacturing technology, used for the transformation of powder to solid state, was selective laser melting, by which the powder material is fully molten, and after its cooling, a final solid structure is created. This process uses powder material which is distributed to the area of the machine bed in a precisely defined thin layer, and the laser beam impacts the area of interest according to the shape of the designed model, which is stored in the STL file. Repeating this process layer-by-layer, the final component of the desired shape is made. Optimal technological parameters were set, due to the reaching of the best possible final structure from the qualitative point of view. The main reason was obtaining the best possible density and the absence of non-designed defects. Parameters were set according to powder manufacturer recommendations and the experiences of the operating staff. They are summarised in Table 2. Specimens were prepared at the Technical University in Ostrava (Czech Republic)—Center of 3D printing Protolab.

**Table 2:** Parameters of experimental specimen preparation.

Parameter	Symbol	Value	Unit
Laser power	*P*	275	W
Scanning velocity	*v*	1150	mm·s^−1^
Hatching distance	*d*	80	μm
Layer thickness	*t_L_*	30	μm
Energy input (1) [22]	*ε*	99.64	J·mm^−1^
Scanning strategy		Stripe (Figure 2)	
Gas protection		Argon (Ar)	

**Figure 2:**
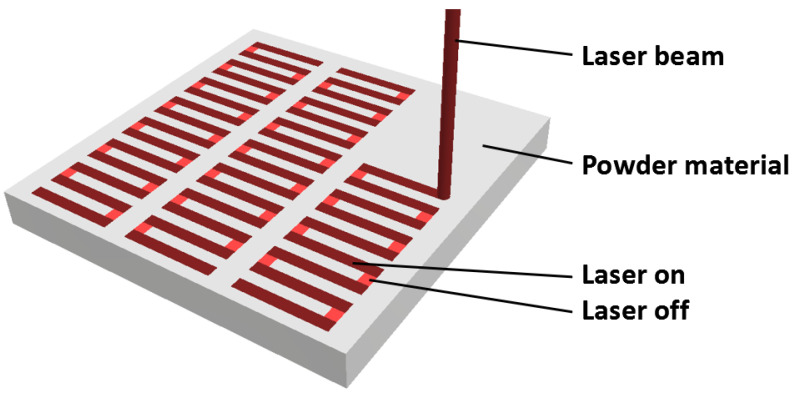
Stripe scanning strategy [23].

*ε* = *P*/(*v* × *d* × *t_L_*) (1)

**Figure 3:**
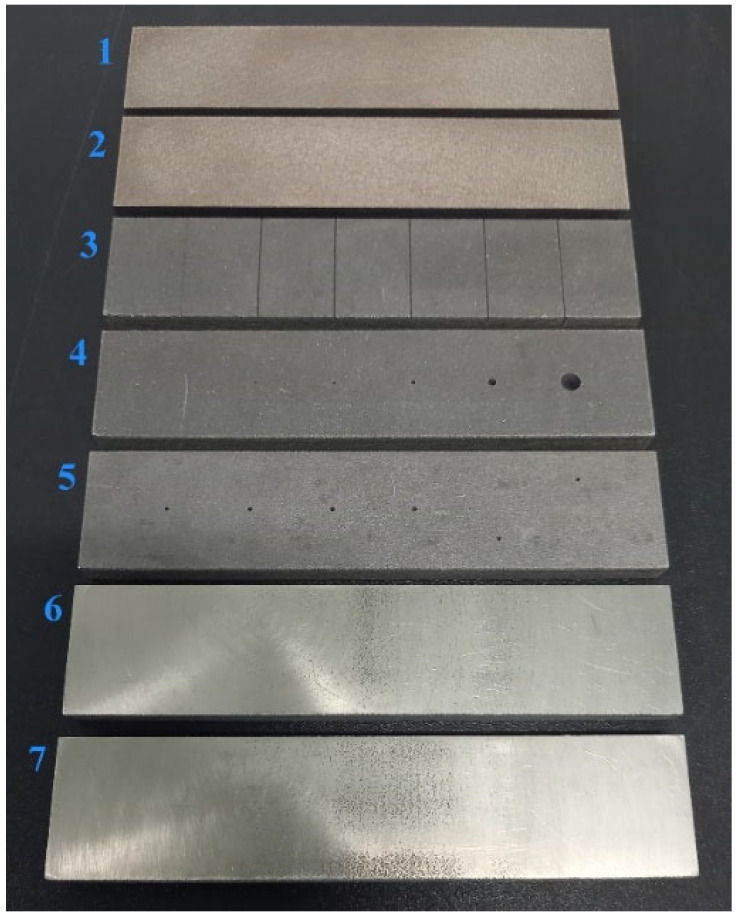
AlSi10Mg experimental specimens with inner rectangular cavities (1 and 2), surface reching defects (3–5) and subsurface spherical cavities (6 and 7).

Specimens were designed with artificial defects in their volume, whose positions, shapes and sizes were adapted to frequently occurring defects during the SLM process. Based on these features, specimens can be divided into four groups. The first group of specimens was designed with rectangular subsurface defects of various sizes and depths, for distinguishing the magnitude of signal deviations as the response to the content of unmolten powder in their volume. In this group, specimen 1 includes 4 cavities of different sizes at 2 mm depth, and specimen 2 includes 3 cavities of the same size situated at various depths and 1 of a different size and in the deepest place. The second group includes specimens with artificial defects reaching their surface layer, with the shape of notches and circular holes. This group includes specimen 3 with 6 notches of various depths on its surface layer and specimen 4, designed with 6 blind holes of various diameters. The third group is represented by specimens including circular holes of various sizes and depths, which are predestined for identification of the surface beyond their rounded bottoms. This group also includes specimen 4, which will be measured from the side of the holes’ rounded bottoms, and specimen 5, including 6 similar blind holes of the same diameter (1 mm) and with their bottoms at different depths. The fourth specimen group involves artificial defects in the form of spherical cavities of various sizes and depths. In this group, specimen 6 is designed with 6 cavities of different sizes at the same depth and specimen 7 with 6 cavities of the same size (1 mm) and at various depths. Overall, 7 specimens were designed (Figure 3), each with dimensions 140 × 30 × 10 mm, and mentioned artificial defects represent real defects in the following manner: thin surface-reaching cracks are represented by notches, hollow pores by rounded bottoms of circular holes and powder-filled pores by spherical cavities. Artificial defects were designed in sufficient mutual distances, for the reason of suppression of the unwanted signal deviations and sufficient distance of specimen edges, because of the edge effect influence. Defects were designed during the creation of CAD models of specimens, intended for subsequent SLM process, and the laser beam created these defects layer-by-layer within specimens, based on the generated STL file.

**Figure 4:**
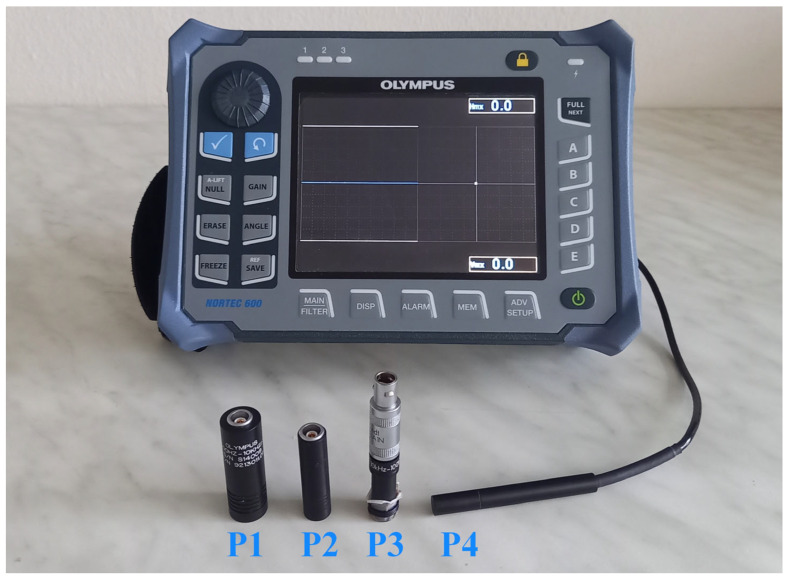
NORTEC 600 eddy current testing device with ECT absolute probes (P1–P3) and conducitivty probe (P4).

For eddy current testing of experimental specimens, the NORTEC 600 portable device by OLYMPUS company (Center Valley, PA, USA) was used (Figure 4). A signal deviation as the response to the presence is rendered by variation in vertical direction (VMAX) and horizontal direction (HMAX), by which the first represents the imaginary part of impedance (reactance), and the second represents its real part (resistance). For various types of artificial defects, various identification modes within a device were selected. The first IMPEDANCE (IMP) mode depicts the signal curves on a vertical and horizontal axis, relative to the middle reference (zero) point. A device also shows maximal signal deviation (VMAX and HMAX) in a numerical expression, recorded within one measurement. The second SWEEP (SWP) mode expresses the signal deviation in the form of signal peaks in vertical direction over time, relative to the horizontal (reference) curve. The third mode allows a combination of both modes. For better interpretation of signal curves, additional functions were adjusted throughout the identification. The GAIN function allows the intensification and attenuation of obtained signal on both axes, and the ANGLE function is intended for rotation of signal curves over 360°. At the application of the SWEEP regime, it is possible to distribute VMAX and HMAX deviations in two separate signal curves, using the AUTO XY MODE [24].

## 3. Results

The expression of basic ECT probe characteristics in the form of reactance/resistance variation, the lift-off effect and the skin effect precedes the identification of the designed artificial defects in AlSi10Mg aluminium alloy experimental specimens.

### 3.1. Evaluation of ECT Probe Characteristics

The possibilities of aluminium alloy identification were evaluated through important parameters of the used eddy current testing probes. Three probes were selected due to the covering of a wide range of excitation frequencies (0.3 to 100 kHz) and consequently ensuring the identification of adequate depth, including the surface and subsurface layers of the evaluated material, for which the ECT method is intended. The selected probes are compatible with the used ECT device, and their capabilities were checked using the investigation of the lift-off effect and standard penetration depth calculation (δ). The fourth conductivity probe was used to obtain the electrical conductivity of the experimental material. The basic parameters of all four probes are summarised in Table 3.

The impedance of the coil, as the main indicator of material variations, can be divided into two main parameters. The reactance is depicted in the form of VMAX, and the resistance is depicted in the form HMAX. The level of reactance and resistance variation (change in VMAX and HMAX) for each probe was monitored at different lift-off values (probe-surface distance), which were reached by the application of non-conductive (plastic) shims between the probe and the surface of a material. Thickness values were 0.15, 0.30, 0.60, 0.90 and 1.20 mm, the probe was attached to the surface of a material, and the values of VMAX and HMAX variation were recorded for the chosen frequencies in the range of individual probes.

#### 3.1.1. Reactance (VMAX)

**Figure 5:**
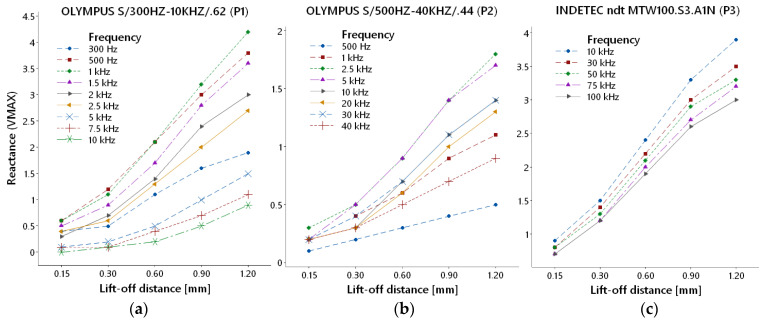
Reactance variation: (**a**) P1 probe; (**b**) P2 probe; (**c**) P3 probe.

Figure 5 includes diagrams of VMAX parameter variations based on increasing lift-off distance for all three probes. In the case of the P1 probe (Figure 5a), nine frequencies were selected. All curves have similar shapes with a growing trend of the VMAX value with the increasing lift-off distance. Less significant graduation occurred in the case of higher frequencies, and the most significant deviations occurred at lower frequencies. The P2 probe (Figure 5b) identification ability was evaluated using eight frequencies. The curves have an increasing character with similar shapes of transition, except for the lowest frequency (500 Hz). Overlapped curves of 2.5 and 5 kHz reached the highest values of VMAX variation. A noticeable increase in reactance (VMAX) at 10 and 20 kHz frequency curves was recorded at the 0.30 mm lift-off distance. These frequencies are from the middle of the P2 probe range, closer to the higher values, and hence, they are not so sensitive to lift-off distance in the whole examined lift-off range like 2.5 and 5 kHz frequencies. However, more significant lift-off variation can cause an increase in their VMAX variation, which is evident in this increase above 0.30 mm distance; hence, they can be useable for greater material changes during measurement. The P3 probe (Figure 5c) was evaluated using five excitation frequencies. All curves have a similar shape with an increasing trend. It can be stated that the deviation grows with the lowering of frequency and growth of lift-off distance. The sensitivity of the probe is more significant in its whole frequency range (VMAX between 3–4 at 1.20 mm) than in the case of probes P1 and P2. The first mentioned probe reached the most significant VMAX deviation (4.3) at 1 kHz frequency and 1.20 mm, and the second one reached the least significant maximal deviation (1.7) of all probes at 2.5 kHz and 1.20 mm.

#### 3.1.2. Resistance (HMAX)

**Figure 6:**
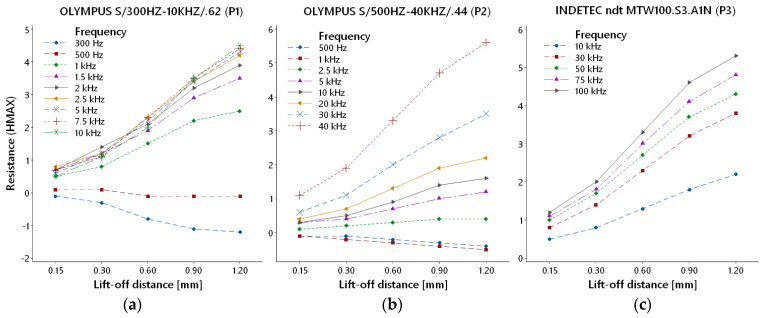
Resistance variation: (**a**) P1 probe; (**b**) P2 probe; (**c**) P3 probe.

HMAX parameter variations based on increasing lift-off distance are summarised in Figure 6 for each probe. The frequency range of all probes was the same as in the case of reactance (VMAX) variation. P1 probe (Figure 6a) curves of most frequencies (1–10 kHz) overlapped each other. All these curves have increasing trends with similar shapes. With the decreasing frequency, HMAX deviation also decreases. At lower frequencies, lift-off distance graduation caused HMAX deviation in a negative sense, with a less steep slope. Figure 6b depicts the resistance variation diagram of the P2 probe, in which frequency curves were more branched. At lower frequencies, curves incline to the negative direction. In the case of probes with higher frequency ranges (P1 and P2), HMAX deviation curves are more branched on the depicted graph due to the higher sensitivity of this parameter to lift-off distance, and it caused the inclination of the mentioned lower frequencies to the negative direction. With the increasing frequency, HMAX deviation increases at the whole transition of other curves. Deviations of the HMAX parameter in the frequency range of the P3 probe are interpreted in the last diagram (Figure 6c). All curves characterise the increasing trend of deviation with the lift-off distance growth. In the case of all three probes, a maximal deviation was reached at their highest excitation frequencies, at a 1.20 mm lift-off distance. For the P1 probe, 10 kHz frequency reached approx. HMAX 4.5, for the P2 probe, 40 kHz reached approx. HMAX 5.7, and for the P3 probe, 100 kHz excitation frequency reached approx. HMAX 5.3.

#### 3.1.3. Lift-off Effect

**Figure 7:**
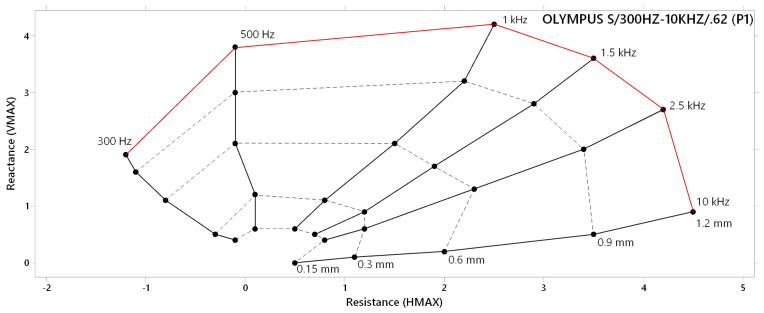
Lift-off effect diagram of P1 probe.

Through the connection of reactance (VMAX) and resistance (HMAX) variations at different frequencies and lift-off distances, a lift-off effect diagram is obtained, which expresses the identification capabilities of the eddy current probe. The next figures include lift-off effect diagrams for each experimental probe, with their typical characteristic shape. For the expression of the lift-off effect related to the P1 probe (Figure 7), six frequencies were selected. It is evident that the difference between VMAX and HMAX is not significant, which causes the balanced shape of the whole diagram. The most balanced deviations are present between curves of 1 kHz and 1.5 kHz, which can be predicted as appropriate for obtaining satisfactory measurement data with the P1 probe. In the case of reaching deeper layers of material, lower frequencies between 300 Hz and 1 kHz are more suitable. Curves of frequencies lower than 500 Hz are shortened as a consequence of losing the sensitivity with decreasing the excitation frequency. On the other side of the diagram, frequencies higher than 1.5 kHz tend to deviate more significantly to the HMAX direction, which similarly causes complicated depiction of identified defects, caused by the higher sensitivity of excitation frequency in its natural manner, but this sensitivity is restricted by the identification ability of the P1 probe at higher (boundary) frequencies. Considering the excitation frequency of this probe (0.3 kHz–10 kHz), the coil is not able to keep consistent sensitivity in its whole frequency range, which is mostly exhibited on boundary frequencies in such types of probes. Hence, it can be stated that the restriction of identification ability can also be caused by the wide frequency range of the probe.

**Figure 8:**
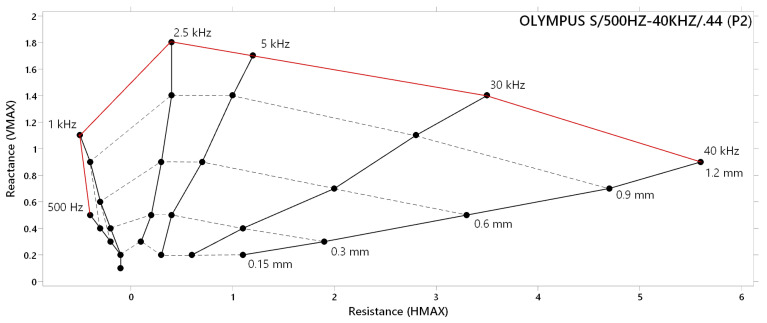
Lift-off effect diagram of P2 probe.

Figure 8 includes a diagram of the lift-off effect related to the P2 probe. Same as in the case of the P1 probe diagram, six frequencies were selected, due to its better readability. The overall shape of a diagram is less balanced, with the wider occupied range on the horizontal axis, as the consequence of approx. three times higher deviation of HMAX variation in comparison with the VMAX parameter. The most balanced deviations of combined VMAX and HMAX parameters are present between 5 kHz and 30 kHz frequency curves, predicted to be suitable for identification. An excitation frequency of 2.5 kHz reached the highest deviation on the vertical axis and can be considered usable, too. In the case of 500 Hz frequency, it can be seen that deviation is negligible, and its application for identification can be restricted. Above the 5 kHz excitation frequency, signal deviations tend to decrease in the vertical direction (VMAX) and increase in the horizontal direction (HMAX) for the whole range of lift-off distances. The most significant decrease occurs between 30 kHz and 40 kHz curves, due to the lower potential of boundary frequencies, caused by the high range of the P2 probe.

**Figure 9:**
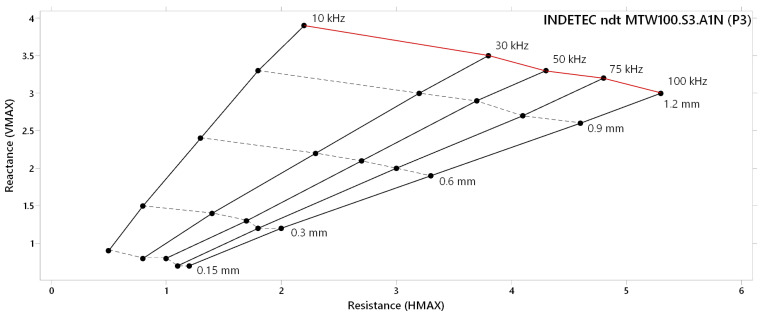
Lift-off effect diagram of P3 probe.

The lift-off effect diagram of the P3 probe is included in Figure 9. Its overall shape can be considered balanced, as the consequence of similar maximal deviations on both axes, with slightly higher deviation of the HMAX parameter at higher frequencies and lift-off distances; thus, predicted sensitivity and consequently identification ability can be similar for the whole frequency range of a probe. The most balanced overall deviation occurred at 30 kHz excitation frequency. At lower frequencies, the deviation of the VMAX parameter increases, and the deviation of the HMAX parameter decreases. Reversibly, the opposite phenomenon occurs with the increasing of excitation frequency, mainly in the case of higher lift-off distances, with its maximum at the 100 kHz curve. According to the lift-off effect diagram of the P3 probe, it can be predicted that its identification potential can be satisfactory at its whole frequency range, but standard penetration depth can be limiting at higher frequencies, which can be decisive.

#### 3.1.4. Skin Effect

**Figure 10:**
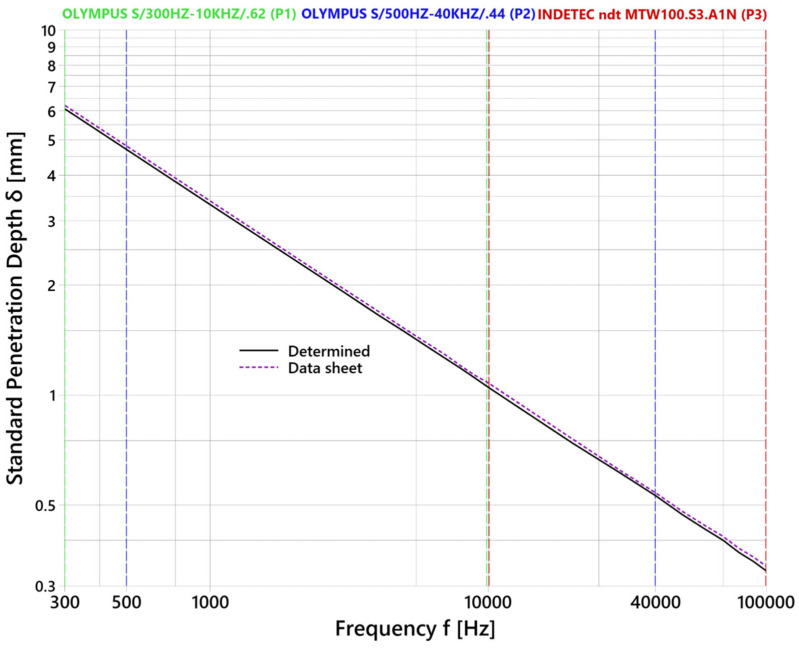
Standard penetration depth diagram.

The skin effect within the ECT expresses the ability of eddy currents to penetrate the material at a certain excitation frequency *f*, with the weakening of its sensitivity. Thus, the mentioned parameter is influenced by coil characteristics (frequency) and material characteristics (magnetic permeability and electrical conductivity). The skin effect is described through standard penetration depth *δ*, where ECT can provide reliable data, and the density of eddy currents is 37% of 100% density on the surface. A depth of penetration is calculated using Equation (2) [1]:

(2) $$\delta =\sqrt{\frac{1}{\pi \times f\times \mu \times \sigma }}$$

where *δ* represents standard penetration depth, *f* is the excitation frequency, *µ* expresses magnetic permeability, and *σ* is the electrical conductivity of the material. The only significant material parameter is electrical conductivity in the case of the AlSi10Mg alloy, because of its non-magnetic character. Its value for the AlSi10Mg material is stated by the manufacturer as 37.76% IACS (International Annealed Copper Standard) at 40 °C [21], which was the main reason for measuring electrical conductivity using the NORTEC 600 device (OLYMPUS company, Center Valley, PA, USA). The experiment was performed at 21 °C, and the conductivity regime was applied after the connection of the OLYMPUS SPO-887L (P4) probe. During the calibration process, the conductivity of low- and high-limit calibration specimens is set. As the low limit, AISI 304 stainless steel was selected, and as the high limit, CW004A copper alloy was selected, with values of 2.39% IACS [25] and 101.42% IACS [26], respectively. Next, a probe was attached to the AlSi10Mg material, and a device calculated its value as 39.52% IACS, which was included in Equation (2). Figure 10 includes a diagram of the standard penetration depth of the AlSi10Mg material. Maximal depth can be reached at 300 Hz frequency (6.07 mm), using the P1 probe. It can measure the material to a minimal depth of 1.05 mm at 10 kHz. It appears as a suitable probe for the identification of subsurface defects. The P2 probe allows identification at frequencies between 500 Hz and 40 kHz, at which the depth is 4.70 and 0.53 mm, respectively. The last P1 probe can reach shallower depths, with a minimal depth of 0.33 mm at 100 kHz and a maximal depth of 1.05 mm at 10 kHz. It could provide more detailed information about identified defects due to its higher sensitivity. The difference between the highest reached depth of data sheet conductivity (6.21 mm) and measured conductivity (6.07 mm) at 300 Hz is 0.14 mm, which decreases with the excitation frequency increasing. At 100 kHz frequency, the depth is 0.34 mm and 0.33 mm, respectively. After the calculation of the *δ* parameter, the deviation is negligible.

### 3.2. Defectoscopy

**Figure 11:**
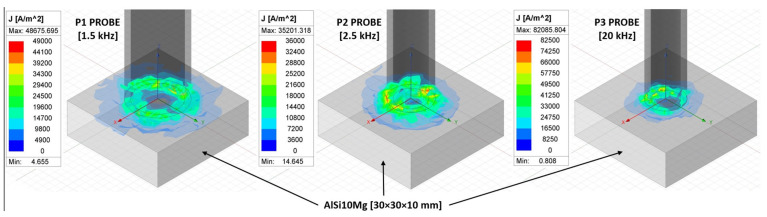
Distribution of eddy currents in material.

The experiment was conducted using the P2 probe, due to its wide frequency range, and the P3 probe, due to its high sensitivity. The P1 probe was excluded because of the considerable influence of the edge effect, which was predicted after testing measurements when the moving of the P3 probe closer to the edge of the specimen caused signal deviations. A distribution of eddy currents in the material was simulated using Ansys Maxwell software 2023 R2. The purpose was not to simulate a real-time measurement but to interpret the possible influence of a larger probe diameter on the edge effect. The main reason is an insufficient amount of information about probe characteristics by manufacturers, important for the creation of the simulation. For the solution, 30 × 30 × 10 mm AlSi10Mg material was used. The current of the coil was set at 20 mA and the number of conductors at 100 for each probe, with diameters related to the used probes. Excitation frequencies were set as the values stated in Figure 11, based on lift-off effect diagrams. It is evident that eddy currents are the least spread in the case of the P3 probe. With the decreasing excitation frequency, the area of material influenced by eddy currents increases. The P1 probe, where eddy currents of minimal density (blue area) reach the edges of the material, could cause the edge effect after the slight movement of the probe further from the middle point and the interaction of denser currents with the edge. The stated values of densities are indicative, and the main factor is probe diameter in combination with excitation frequency.

Measurement device parameters were adjusted based on the characteristics of probes and defects themselves. They are summarised at the end of the section, same as the signal deviations for each defect within each specimen.

#### 3.2.1. Areas with Unmolten Powder

**Figure 12:**
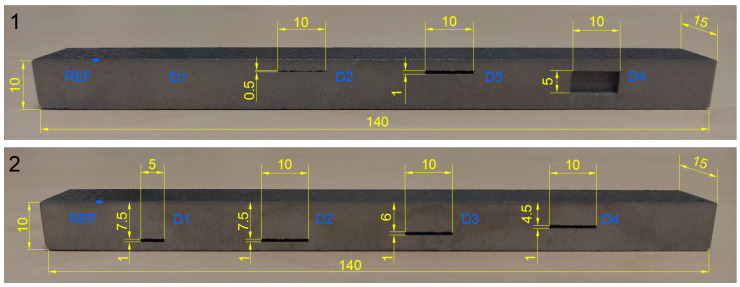
Rectangular cavities (5 mm wide) in specimens 1 and 2 (cross-section) [mm].

**Figure 13:**
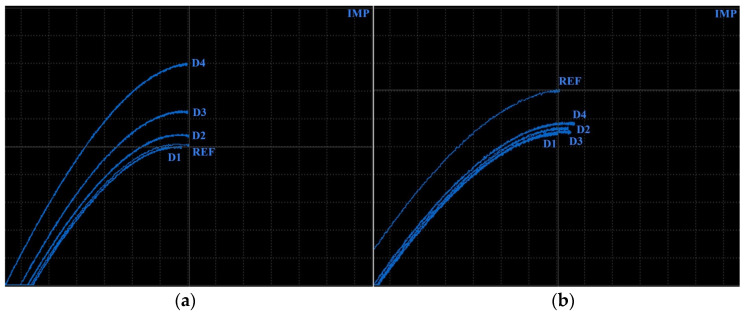
ECT signal variation: (**a**) specimen 1; (**b**) specimen 2.

Specimens 1 and 2 were designed with inner defects in the form of cavities of the rectangular cross-section, in various sizes at the same depth of their upper face (Specimen 1—Figure 12) and vice versa in similar sizes at various depths (Specimen 2—Figure 12). The main purpose was to monitor the impedance signal deviation caused by the eddy current density variation and influenced by different densities of unmolten material. The P2 probe was selected, and identification itself was performed in the form of attaching the probe to the surface layer of specimens in the places of defects in their middle point. Signal deviations were compared with the reference (zero) signal of the reference point in the place of fully molten powder material; thus, the signal curves of defects were evaluated considering the reference signal curve in the center of the cross on the screen. Both specimens were identified using the IMP regime at 2.5 kHz. For better resolution between signal curves, V GAIN, H GAIN and ANGLE functions were adjusted. Also, the reference center of the cross was moved to a 70% position on the vertical axis. It is evident that variation of the influenced eddy current density by various unmolten material volumes causes impedance signal change, whereas its deviation grows with the increase in the unmolten powder amount, according to signal curves in Figure 13a. All artificial defects of specimen 1 were at a 2 mm depth. A D1 defect of 0.2 mm thickness was not created during the SLM process and hence did not cause any deviation, and the curve slightly overlapped the curve of the reference signal. The increasing size of cavities (D2, D3) caused signal curves to distance from the REF point, and the D4 defect signal curve reached the highest deviation, equal to 2.9 VMAX and −0.1 HMAX values. During the measurement of specimen 2, signal deviation curves occupied a close area on the screen (Figure 13b), which is the consequence of the similar shape of cavities. The highest deviation of the VMAX parameter was caused by the D1 defect (−1.4), even though its size is the smallest, and its presence is the deepest. The D4 defect caused the lowest VMAX deviation (−1.2) and the highest HMAX deviation (0.7). Nevertheless, it can be stated that differences between individual deviations are negligible. A more influencing parameter on the impedance variation is its size than the depth of presence. In the case of increasing the size of the defect (specimen 1), the VMAX parameter varied more significantly, and in the case of varying the depth of presence (specimen 2), the HMAX parameter was more influenced. At the used frequency (2.5 kHz), a standard penetration depth is 2.10 mm. Considering the position of defects, a probe was able to observe material changes below this depth. However, measurement data could be distorted, or it can be predicted that the ECT method can identify the material at greater depths.

#### 3.2.2. Surface-Reaching Artificial Defects

**Figure 14:**
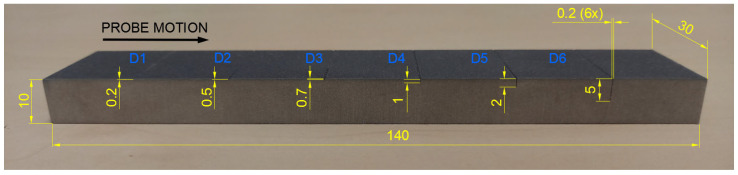
Notch-type artificial defects in specimen 3 [mm].

**Figure 15:**
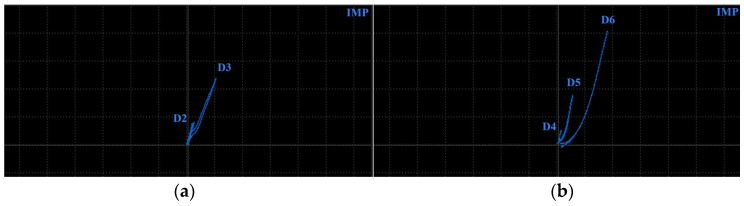
ECT signal variation in specimen 3: (**a**) D2 and D3 defects; (**b**) D4–D6 defects.

Surface-reaching defects, occurring in SLM materials, include mainly thin cracks and small pitting/craters. It is appropriate to use higher excitation frequencies for better depiction of the obtained signal curves; hence, the P2 and P3 probes have predispositions for this purpose. Two specimens were designed. Specimen 3 includes six notches on its surface layer of 0.2 mm width, representing the mentioned cracks, which propagated through the whole width of the specimen. Each notch had a different depth (Figure 14), and the significance of ECT signal deviation was evaluated according to this variable. During the measurement, the probe was moved through the central axis on a surface layer of the specimen, in the longitudinal direction, with the application of the IMP regime. The P2 probe was used for notches of greater depths (D4–D6) at 2.5 kHz (Figure 15b). The most deviated curve was obtained during the measurement of the deepest notch (D6), in the form of a peak with a stronger deviation of VMAX, equal to 4, which is approx. double that of HMAX (1.8). The mentioned VMAX and HMAX differences are present in the case of all notches. The decrease in overall signal deviation occurred with the reduction in their depth. Considering that smaller notches will reach lower signal variation, D1–D3 were identified using the P3 probe at 20 kHz. The identification output screen (Figure 15a) includes a curve of D2 and D3 notches, by which the second caused higher impedance deviation (2.4 VMAX and 1.1 HMAX). D1 was excluded from the identification, due to the very low signal variation, whose curve was lost in the noise signal.

**Figure 16:**
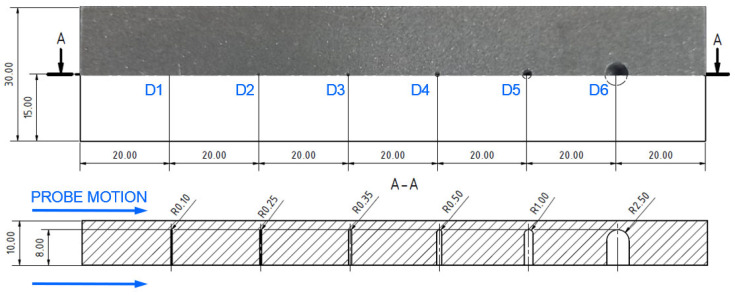
Blind hole-type artificial defects in specimen 4 [mm].

**Figure 17:**
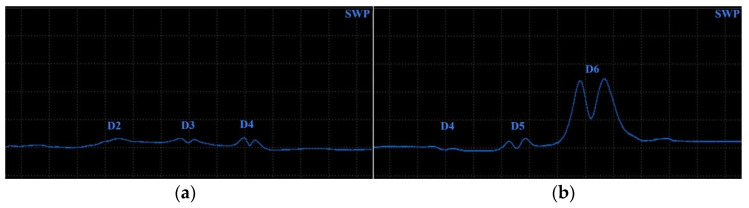
ECT signal variation in specimen 4 (surface): (**a**) D2–D4 defects; (**b**) D4–D6 defects.

Specimen 4 was designed with six blind holes of different diameters with the same depth, situated on the center axis of the specimen in the longitudinal direction (Figure 16). During the identification, holes were measured from the side reaching the surface. Figure 17 includes the measurement device output of identification specimen 4 using the SWP regime. For bigger holes (D4–D6), the P2 probe was applied on 2.5 kHz (Figure 17b). The obtained signal deviation curves are characterised by a double peak, which is related to the defects of circular shapes. A decrease in the signal curve in its middle can be caused by the lower sensitivity of the probe when the identified defect is under its center axis, where eddy currents are slightly suppressed. The highest deviation was caused by the largest hole (D6) on values 4 VMAX and 1.7 HMAX. A deviation decreases with the diameter of the hole, and the curve of the D4 defect is very low and hence could be lost in the noise signal. For this purpose, D4 was included in the identification of smaller holes, using the P3 probe at 20 kHz frequency (Figure 17a). Here, the smallest hole (D1) caused no impedance variation. Other curves resemble a double-peak shape, except the D2, where the signal curve is in the form of a single peak.

**Figure 18:**
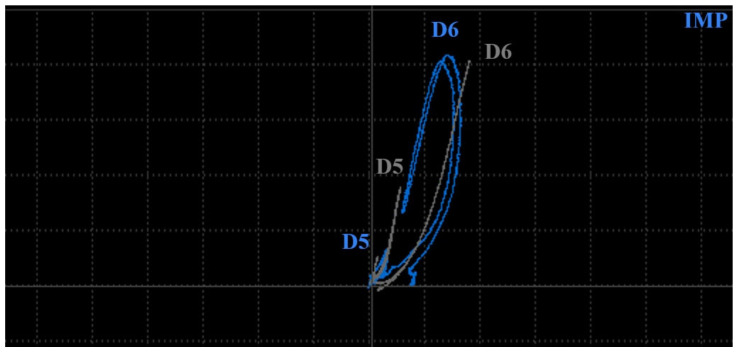
Comparison of ECT signal variation (IMP regime) in specimens 1 and 2 (D5 and D6 defects).

Figure 18 includes a comparison of the signal within the measurement of D5 and D6 holes in specimens 1 (grey curves) and 2 (blue curves) using a P2 probe at 2.5 kHz excitation frequency and IMP regime. Numerical deviations at D6 defects are similar (4 VMAX and 1.7 HMAX); however, the shape is different.

**Figure 19:**
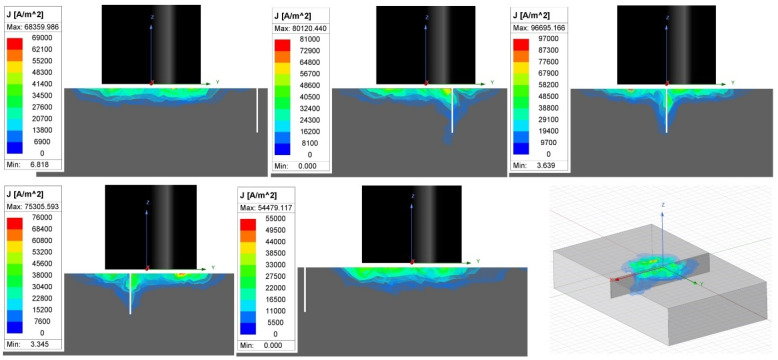
Simulation of eddy currents interaction with notch-type defect.

A transition of the D6 signal curve in specimen 1 starts from the zero point mainly to the vertical direction during the first interaction of eddy currents with a notch. During the next motion, the notch crosses its diameter, and the highest density of eddy currents is influenced; hence, the peak is reached. Next, the described progress is reversed, and the signal is transited to the zero through the same trajectory (Figure 18). The described scenario is interpreted in Figure 19 using the simulation. The configuration of the P2 probe and material was equivalent to the simulation parameters described at the beginning of Section 3.2, with 2.5 kHz and the designed notch (D6 defect) in specimen 3. The values of eddy current density are indicative, but their growing and descending trend based on the probe position confirms the described development of the curve trajectory.

**Figure 20:**
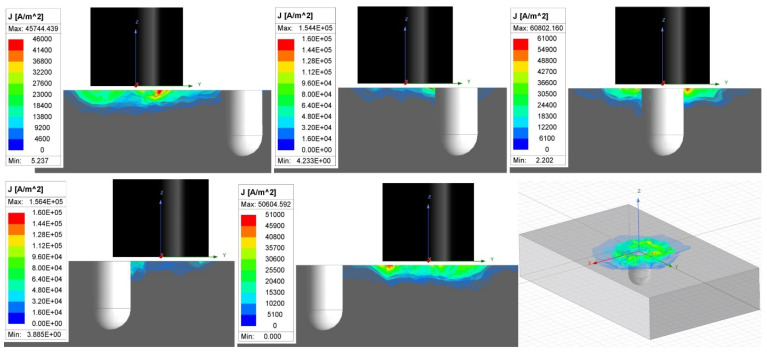
Simulation of eddy current interaction with hole-type defect.

In the case of the D6 hole in specimen 2, the signal curve passes from the zero point mainly to the vertical direction on the rounded trajectory, caused by the rounded shape of the defect. The highest deviation is reached in front of the middle point of the probe, where eddy currents are mostly influenced. Next, the curve trajectory tends to be directed toward the zero point, due to the weakening of eddy currents in the area around the center of the probe. The peak is reached when the middle points of the probe and hole are coaxial. The zero is not reached because the hole causes a certain density variation of eddy currents. Next, a reversed phenomenon occurs, and the curve transits back to the area of the zero signal (Figure 18). The scenario is interpreted in Figure 20. The parameters were similar to the previous, but the designed defect is the hole D6 in specimen 4, and again, the variation of eddy current density confirms the mentioned signal curve development.

The same scenario can be described in the case of D5 defects in both specimens but with a lower magnitude of signal deviation, mainly in the case of specimen 2, where deviation on the vertical axis is approx. a third of specimen 1 (Figure 18).

#### 3.2.3. Subsurface Artificial Defects Unfilled with Powder

**Figure 21:**
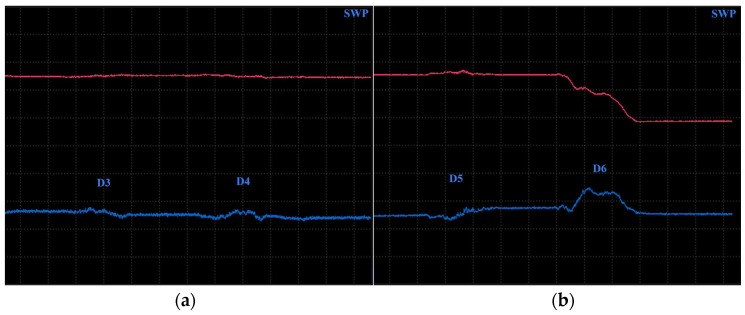
ECT signal variation in specimen 4 (subsurface): (**a**) D3 and D4 defects; (**b**) D5–D6 defects; primary evaluated curves—blue; secondary curves after AUTO XY filtration—red.

Subsurface defects, arising during the SLM process are commonly in the form of pores or cavities. For this purpose, the previously described specimen 4 (Figure 16) was measured from the surface below the rounded bottom of the designed holes. A P2 probe at lower excitation frequency was selected, using the SWP regime. Also, AUTO XY mode was applied for the filtration of the signal on both axes. Wider holes (D5, D6) were identified at 1.5 kHz (Figure 21b). The D6 hole was reliably identified with numerical values 1.7 VMAX and 2 HMAX, depicted by a double peak curve. At the D5 hole, the signal curve is not unequivocal. A similar phenomenon occurred during the measurement of smaller holes (D3, D4), when lower frequency (1 kHz), higher HGAIN (90 dB) and VGAIN (80 dB) were set, which consequently led to a higher noise signal throughout the curve (Figure 21a). Signal deviations are slight, and the identification of similar defects could be more challenging. No significant signal deviations were recorded in the case of holes D1 and D2.

**Figure 22:**
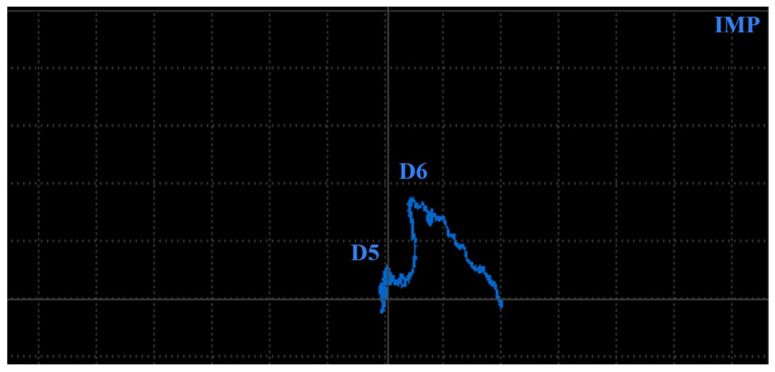
ECT signal variation (IMP regime) in specimen 4 (subsurface): D5 and D6 defects.

Since hole D6 caused considerable signal deviation, the IMP regime was tested using the same parameters (Figure 22). A signal curve of the D6 hole is reliably rendered in the form of the peak, unusual for such type of defect. A transition of the curve starts from the zero position straight forward in a vertical direction to its peak. After moving the probe from its center, a curve decreases in a vertical direction to zero along with increasing in a horizontal direction, which is caused by the setting of high signal intensification using GAIN and the level of signal rotation using ANGLE. A deviation of the D5 hole renders a curve of a similar shape but of a much smaller size, which is difficult to identify, due to its loss in the noise signal, especially during measurement at such high signal intensification.

**Figure 23:**
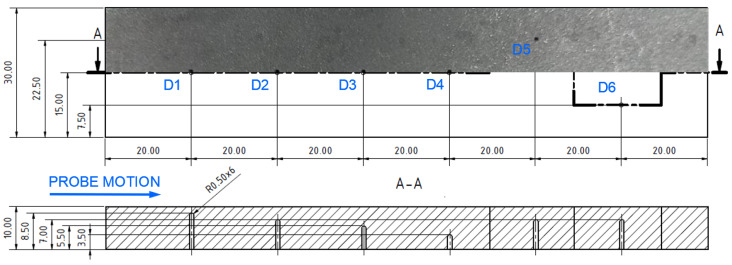
Blind-hole-type artificial defects in specimen 5 [mm].

**Figure 24:**
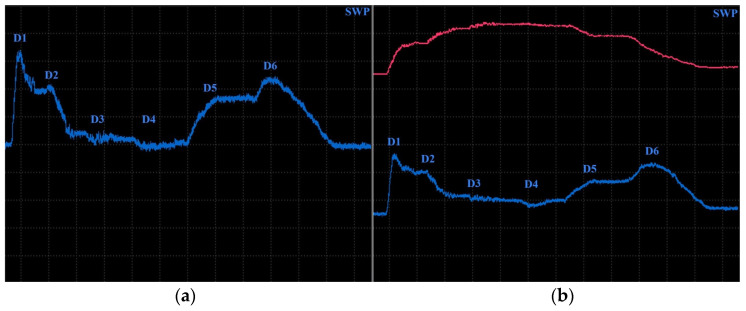
ECT signal variation in specimen 5: (**a**) without AUTO XY; (**b**) with AUTO XY; primary evaluated curves—blue; secondary curve after AUTO XY filtration—red.

Specimen 5 was designed to analyse impedance signal deviation according to various depths and positions of hole-type defects (similar to specimen 4) of the same radius (0.5 mm—Figure 23). Four defects (D1-D4) were designed with various depths, situated on the center axis in a longitudinal direction, and two defects (D5, D6) were situated at a D2 defect’s depth but 7.5 mm from the center in both directions of the transverse axis. The P2 probe was selected, using the same parameters as in the case of the D4 defect in specimen 4, except the ANGLE function. The next figure interprets SWP regime output without (Figure 24a) and with (Figure 24b) the AUTO XY filtration. The most significant deviation was caused by the hole nearest to the surface. With the increasing depth, a decrease in signal deviation occurs. D3 and D4 holes caused negligible signal variation with a slight signal noise increase. A reason can be the position of these defects below the standard penetration depth (3.32 mm). During the measurement of D5 and D6 defects, a deviation increased to the level approximately of the D2 defect. The shape of the signal curve after the application of AUTO XY mode has the same transition, but it is less burdened by the noise signal.

#### 3.2.4. Subsurface Artificial Defects Filled with Powder

**Figure 25:**
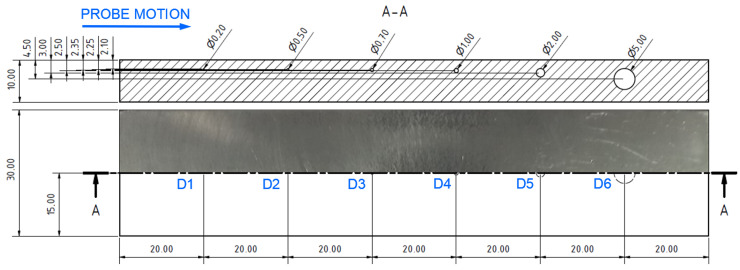
Sphere-type artificial defects in specimen 6 [mm].

**Figure 26:**
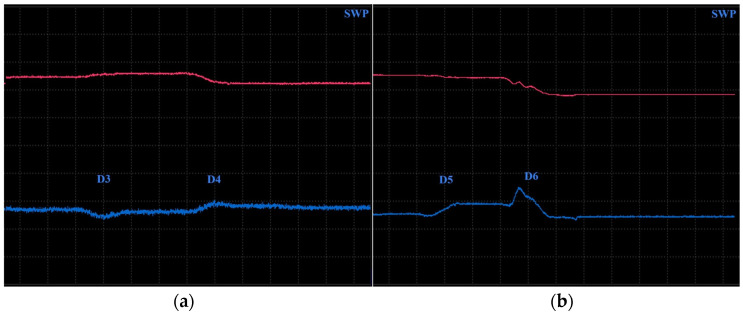
ECT signal variation in specimen 6: (**a**) D3 and D4 defects; (**b**) D5 and D6 defects; primary evaluated curves—blue; secondary curves after AUTO XY filtration—red.

In certain cases, pores and cavities in SLM material can be filled by the unmolten powder, which can influence the magnitude of eddy current density. For such identification ability check, specimen 6 including six spherical cavities was designed. They are in the same dimensions and positions as the artificial defects of specimen 4, with their top points 2 mm below the identified surface layer (Figure 25). A measurement ran identically to other specimens, using the P2 probe. Figure 26 depicts the measurement output using the SWP regime with AUTO XY filtration. Cavities D5 and D6 were identified at excitation frequency, equal to 1.3 kHz (Figure 26b). D3 and D4 cavities were identified at 0.9 kHz frequency, with considerably higher intensification (VGAIN–94 dB and HGAIN–84 dB) (Figure 26a). Traditionally, a signal deviation decreased with a decrease in cavity size; hence, the most significant variation occurred at the D6 defect. D1 and D2 cavities were not identified during the measurement. No defect caused the typical double-peak shape of a curve, because of significant signal attenuation, as the consequence of the presence of material between the probe and the defect, in combination with residual powder in a cavity. It is evident that defects with such characteristics could be difficult to identify in the analysed SLM aluminium alloy.

**Figure 27:**
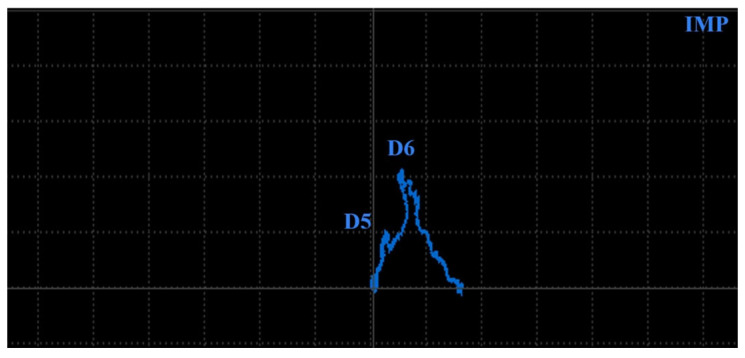
ECT signal variation (IMP regime) in specimen 6: D5 and D6 defects.

Figure 27 includes the interpretation of signal variation during the measurement of larger defects using the IMP regime. The D6 cavity caused a signal deviation mainly on the vertical axis (2.1 VMAX) with a similar curve transitions to its peak during the coincidence of the probe and defect axis and back to the zero point on the vertical axis. The shape of the D5 curve is similar but with a smaller size, which could be difficult to read during the measurement of a larger number of such defects. The untypical shape of both curves can be the consequence of similar factors described during the identification of D5 and D6 defects in specimen 4 using the IMP regime (Figure 22).

**Figure 28:**
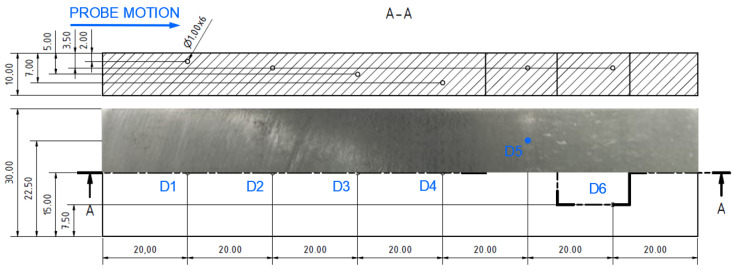
Sphere-type artificial defects in specimen 7 [mm].

**Figure 29:**
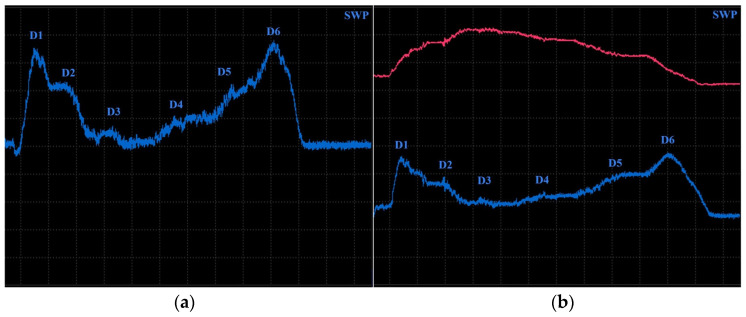
ECT signal variation in specimen 7: (**a**) without AUTO XY; (**b**) with AUTO XY; primary evaluated curves—blue; secondary curve after AUTO XY filtration—red.

Specimen 7 was designed with cavities of 1 mm in diameter at various depths and positions. Same as in the case of specimen 5, D1–D4 cavities were situated at different depths on the center axis in a longitudinal direction. D5 and D6 cavities were 7.5 mm from the center axis in both directions of the transverse axis (Figure 28). The P2 probe was selected using the SWP regime without (Figure 29a) and with the setting of AUTO XY mode (Figure 29b). Other parameters were used according to the measurement of the D4 defect in specimen 6, with lower signal intensification. The overall signal curve shape is similar to specimen 5 but with more significant noise in both interpretations. The strongest peak was reached during D1 cavitation, with a following decrease, as the consequence of a deeper situation of defects, whereas the signal of D3 and D4 cavities can be considered as lost in the noise signal. D5 caused deviation similar to D2 and D6 similar to D1, which can be ascribed to their similar depth of presence. However, such identification results are complicated to analyse.

Table 4 summarises the parameter setting of the ECT device within the measurement of each specimen. The P2 probe was used in a majority of defects, except for smaller surface-reaching defects, where the P3 probe was used. The IMP regime was preferred for specimens 1 and 2, surface-reaching defects and defects of larger sizes. Excitation frequencies in the range of 0.9–2.5 kHz were applied based on the character of defects, and the highest frequency was 20 kHz. GAIN parameters were adjusted to higher values for smaller defects and higher depths. ANGLE was set at suitable values based on the depicted curves on the screen. The maximum calculated penetration depth was 3.50 mm at 0.9 Hz frequency.

Table 5 includes numerical expressions of signal deviations during the investigation of all specimens. The values of VMAX and HMAX are expressed separately for each identified artificial defect. In the case of specimens 1 and 2, measurements were repeated three times, and an average value was expressed. A deviation of D4 in specimen 4 includes two values. The first is related to identification within the measurement of smaller defects in specimen and the second within the measurement of larger defects.

## 4. Discussion

Although it is difficult to compare the obtained results due to the absence of similar studies on the identification of defects in the AM AlSi10Mg aluminium alloy, certain parts of the described experiment can be compared with other experiments in the field, which were conducted on the same material or with a similar purpose on different materials. A study [17] focused on the relative density measurement of AlSi10Mg material showed its significant influence on the electrical conductivity of the material and consequently on the ECT device signal. A similar phenomenon was described during the measurement of specimen 1, where various signal deviations were obtained in areas with rectangular cavities of various sizes including unmolten powder, thus with different densities of material. Also, the graduation of signal deviation with increasing sizes of defects in other specimens confirms this fact. A measurement of electrical conductivity using an ECT device in research [20], focused on the heat treatment of the DMLS AlSi10Mg alloy, obtained a conductivity of 28.5% IACS of the as-built state, 43.6% IACS after stress-relieving heat treatment and 44.2% IACS after T6 heat treatment. For the material in the presented experiment, the manufacturer states an electrical conductivity of 37.76% IACS at 40 °C, and the experimentally obtained value is 39.52% IACS. Such differences in conductivity values can be ascribed to different types of additive manufacturing processes and powder material composition. It is an important indicator of the necessity of electrical conductivity measurement for the evaluated material during the preparation of the experiment, due to its sensitivity to various material states and environmental temperature. Taking the shape of signal curves based on the shape of the defect into account, analysis in a study carried out on AM stainless steel and titanium alloy [14] pointed to the single peak obtained at the notch-type defect and double peak at the sphere-type defect, which was also found in the presented experiment; thus, it can be stated that the ECT signal has certain characteristics at certain types of defects, and it does not matter in what material it is present. Nevertheless, the magnitude of the curve on both axes can be influenced by material, due to its different influence on the attenuation of the ECT signal (Table 6). The smallest detected inner defect was 0.7 mm in diameter, which is bigger than in other examined AM materials by other authors, including Inconel 738LC alloy (0.4 mm) [16], Co-based alloy (0.5 mm) [27] or SS 316L stainless steel and titanium alloy Ti64 (0.2 mm) [14]. The maximal reached depth is superior, which was 3.5 in this experiment, in comparison to 1.1 mm [16] and 1 mm [27], with the exception of 3.5 mm [14]. However, depth is also influenced by the designed artificial defects, evaluated material, used probe and excitation frequency.

## 5. Conclusions

The experiment described here is focused on the eddy current testing method and checking its abilities in the identification of aluminium alloy AlSi10Mg prepared by selective laser melting technology. The fundamental characteristics (lift-off effect and skin effect) of three selected probes are evaluated, and eddy current signal deviations based on the designed artificial defects are analysed, with subsequent expression of optimal measurement parameters. The obtained data are related to the used probes and devices, but certain findings can be helpful for subsequent research focused on the ECT of evaluated material and alternatively for the design of specific probes with better properties. Based on the collected data, as the output of the experimental procedure, key conclusions can be formulated:

- The lift-off effect diagrams of selected probes for the AlSi10Mg alloy were expressed, with a prior definition of reactance (VMAX) and resistance (HMAX) variation dependent on probe-surface distance.
- Electrical conductivity as an important material parameter was determined for the AlSi10Mg alloy using an ECT device, with subsequent calculation of standard penetration depth in the frequency range of the used probes (0.3–100 kHz).
- The IMPEDANCE regime was shown to be applicable solely for surface-reaching defects and possibly for larger subsurface defects (5 mm in diameter). Also, a regime was used for distinguishing signal variations based on the presence of areas with various densities of unmolten powder (specimen 1). For other types of defects, a simpler SWEEP regime is suitable, and hence, a lower level of data interpretation is reached.
- Maximal reached depth was 3.50 mm at 0.9 kHz frequency, according to standard penetration depth calculation. D4 defects (1 mm diameter) at 6.5 mm in depth of specimens 5 and 7 caused slight signal deviation; however, its magnitude is negligible and hard to analyse, which confirms difficulties in the identification of defects in the subsurface layers of AlSi10Mg material.
- The smallest detected surface-reaching defects were 0.5 mm in diameter (hole type) and at 0.5 mm in depth (notch type). For subsurface defects, the smallest detected diameter was 0.7 mm, in the case of both types, empty holes and spherical cavities filled by unmolten powder, with a slight signal deviation and setting of higher values of the GAIN parameter mainly in the case of the second one mentioned.
- The dependency of signal curves based on defect characteristics was expressed in the form of a single peak for the notch-type defect in both regimes, a double peak for spherical defects within the SWP regime and a wave-type curve for the IMP regime. A curve development for these types was described with simulation interpretation.

Even though some important findings were concluded from the presented experiment, it is necessary to use and develop the obtained preliminary results within subsequent experiments before the creation of a comprehensive methodology. During the further direction of research, it is important to test the ECT method for more complicated shapes of AlSi10Mg material, because AM components are not always simple. Furthermore, defects can occur in more complex shapes and in clusters, whose detectability using the ECT method needs to be checked, with subsequent application of a method for real defects and comparison with results obtained using X-ray tomography. Consequently, it is necessary to check the ability of the ECT method on other frequently used AM materials, within the creation of methodology.

## Figures and Tables

**Table 1:** Chemical composition of AlSi10Mg aluminium alloy [21].

Element	Al	Si	Fe	Cu	Mn	Mg	Ni	Zn	Pb	Ti	Sn
Mass (%)	Balance	9–11	≤0.55	≤0.05	≤0.45	0.2–0.45	≤0.55	≤0.1	≤0.05	≤0.05	≤0.05

**Table 3:** ECT probes used for experiment.

Label	Probe	Type	Diameter [mm]	Frequency Range [kHz]
P1	OLYMPUS S/300 HZ-10 KHZ/.62	Absolute	15.7	0.3–10
P2	OLYMPUS S/500 HZ-40 KHZ/.44	Absolute	11.2	0.5–40
P3	INDETEC ndt MTW100.S3.A1N	Absolute	10	10–100
P4	OLYMPUS SPO-887L	Conductivity	7.9	60

**Table 4:** ECT device parameters.

Specimen	Defects	Probe	Regime	*f* [Hz]	VGAIN [dB]	HGAIN [dB]	ANGLE [°]	*δ* [mm]
1	D1–D4	P2	IMP	2500	82	62.6	28	2.10
2	D1–D4	P2	IMP	2500	83.5	64.5	30	2.10
3	D2–D3	P2/P3	IMP	20,000	58	58	120	0.74
	D4–D6	P2	IMP	2500	63	63	133	2.10
4 (surface)	D2–D4	P2/P3	SWP	20,000	65	55	20	0.74
	D5–D6	P2	SWP/IMP	2500	68.4	70.7	133	2.10
4	D3–D4	P2	SWP	1000	90	80	206	3.32
	D5–D6	P2	SWP/IMP	1500	84	73	182	2.71
5	D1–D6	P2	SWP	1000	90	80	225	3.32
6	D3–D4	P2	SWP	900	94	84	225	3.50
	D5–D6	P2	SWP/IMP	1300	84	73	192	2.92
7	D1–D6	P2	SWP	900	92.5	82.5	225	3.50

**Table 5:** ECT signal deviations.

Specimen	VMAX						HMAX					
	D1	D2	D3	D4	D5	D6	D1	D2	D3	D4	D5	D6
1	0	0.6	1.3	2.9	-	-	−0.3	0	0	−0.1	-	-
2	−1.4	−1.2	−1.3	−1.1	-	-	0	0.5	0.6	0.7	-	-
3	-	0.6	2.4	0.5	1.8	4	-	0.1	1.1	0.1	0.5	1.8
4 (surface)	-	0.4	1.1	1.9/0.1	0.7	4	-	0.1	0.6	1.0/0.1	0.3	1.7
4	-	-	−0.5	−0.8	0.5	1.7	-	-	−0.3	−0.3	0.4	2
5	3.5	2.1	1.2	0.5	1.8	2.9	−1.8	−3.3	−4.1	−4.1	−3.6	−2.2
6	-	-	0.5	1.2	0.9	2.1	-	-	0.2	0.7	0.3	1.6
7	3.4	0.6	−0.2	−0.1	1	3.7	−1.4	−2.6	−3.4	−3	−1.9	−0.5

**Table 6:** Comparison list of results with other materials.

Material	Smallest Defect [mm]	Reached Depth [mm]
AlSi10Mg	0.7	3.5
SS 316L [14]	0.2	3.5
Ti64 [14]	0.2	3.5
Inconel 738LC [16]	0.4	1.1
Co-based alloy [27]	0.5	1

## Data Availability

Data are contained within the article.
